# Immunological cross-talk between periodontitis and systemic lupus erythematosus: the role of T–B cell dysregulation in the shared pathogenic network

**DOI:** 10.3389/fimmu.2026.1863200

**Published:** 2026-09-29

**Authors:** Weiwen Hu, Jianxiong Wang, Jingjing Tong, Zhijia Xu, Weile Chen, Rui He, Wei Wei, Shenqian Xu, Xiaoyu Sun, Jiajie Tu

**Affiliations:** 1 Institute of Clinical Pharmacology, School of Pharmacy, Anhui Medical University, Key Laboratory of Anti-inflammatory and Immune Medicine (Anhui Medical University), Ministry of Education, Hefei, China; 2 Department of Rheumatology & Immunology, The First Affiliated Hospital of Anhui Medical University, Hefei, China; 3 College & Hospital of Stomatology, Anhui Medical University, Anhui Province Key Laboratory of Oral Diseases Research, Hefei, China

**Keywords:** adaptive immune dysregulation, BAFF, IL-21, periodontitis, systemic lupus erythematosus, T–B cell crosstalk

## Abstract

Periodontitis and systemic lupus erythematosus (SLE) exhibit overlapping immunopathological features characterized by dysregulated adaptive immune responses. Emerging evidence suggests that aberrant T–B cell crosstalk represents a potential immunological link between chronic oral inflammation and systemic autoimmunity. Persistent periodontal inflammation promotes the release of pathogen-associated molecular patterns (PAMPs) and damage-associated molecular patterns (DAMPs), leading to dendritic cell activation, neutrophil extracellular trap (NET) formation, and sustained immune stimulation. These signals may facilitate abnormal germinal center responses through enhanced Tfh–B cell interactions and increased BAFF and IL-21 signaling, thereby amplifying B-cell activation, plasma-cell differentiation, and antibody production. Conversely, SLE-associated systemic immune dysregulation may aggravate periodontal inflammation, suggesting a potentially bidirectional interaction between oral and systemic immunity. However, most available evidence has been obtained from separate studies of periodontitis and SLE and does not establish a shared causal pathway between the two diseases. In this review, we summarize current evidence supporting the role of T–B cell dysregulation as a potential immunological link between periodontitis and SLE. We further discuss candidate mechanisms involving dendritic cells, NETs, BAFF, IL-21, and germinal center reactions, as well as their therapeutic implications. Elucidating the oral–systemic immune axis may provide novel insights into integrated immunomodulatory strategies for autoimmune diseases.

## Graphical Abstract

**Figure:**
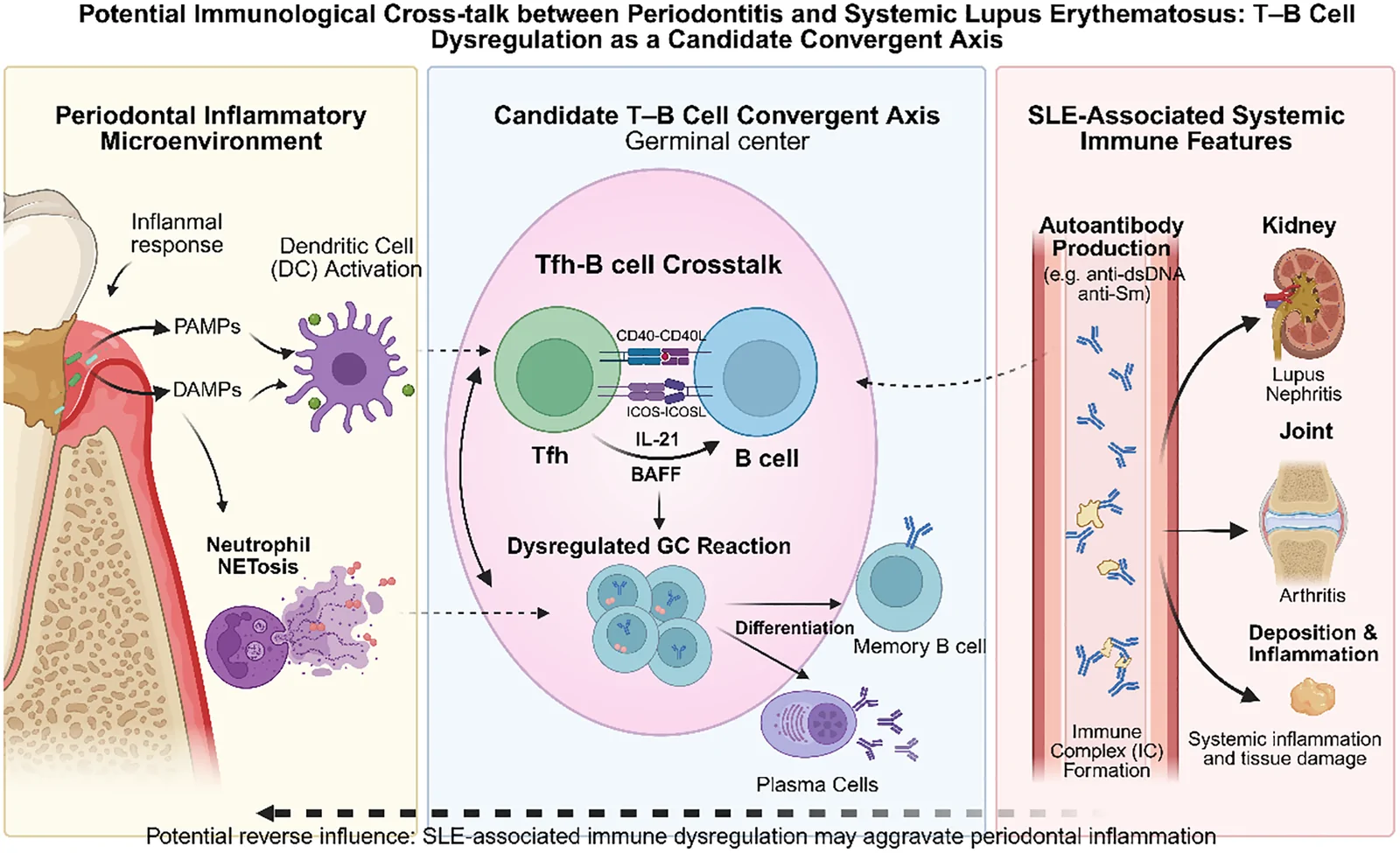
Potential immunological crosstalk between periodontitis and SLE centered on T–B-cell dysregulation. Periodontal inflammation is associated with PAMP/DAMP release, dendritic-cell activation, and NET formation, processes that may converge with altered Tfh–B-cell signaling involving CD40–CD40L, ICOS–ICOSL, IL-21, and BAFF. In susceptible individuals, these pathways could contribute to enhanced B-cell activation and humoral autoimmunity. Conversely, SLE-associated systemic immune dysregulation may aggravate periodontal inflammation. Solid arrows denote well-supported within-disease relationships, whereas dashed arrows indicate proposed cross-disease links that remain to be directly validated. This schematic represents a hypothesis-generating framework and does not establish causality.

## 1 Introduction

Systemic lupus erythematosus (SLE) is a systemic autoimmune disease characterized by loss of immune tolerance, dysregulated T and B cell responses, and persistent production of pathogenic autoantibodies, ultimately leading to multiorgan damage (1). Although genetic susceptibility and intrinsic immune abnormalities constitute the core pathogenesis of SLE, environmental and mucosal factors that may trigger or sustain systemic immune activation have attracted increasing attention in recent years (2).

Periodontitis is a highly prevalent chronic inflammatory disease initiated by dysbiosis of the subgingival microbiota and progresses due to persistent host immune responses (3). Several epidemiological studies, systematic reviews, and nationwide cohort analyses have reported a higher prevalence or risk of periodontitis among patients with SLE than among controls (4–6). Moreover, multiple observational studies have found that the severity of periodontitis may correlate with SLE disease activity. Preliminary interventional studies have shown that periodontal therapy may reduce systemic inflammatory markers and disease activity scores (6). However, the available evidence is predominantly observational or derived from small interventional studies and is potentially affected by medication use, smoking, oral hygiene, and disease severity; therefore, it does not establish a causal relationship. Conversely, SLE-associated systemic immune dysregulation and its treatment may also influence periodontal susceptibility and severity.

From an immunological perspective, periodontitis and SLE exhibit overlapping features, including persistent activation of innate immunity, breakdown of immune tolerance, expansion of proinflammatory T-cell subsets, aberrant survival and differentiation of B cells, and amplification of the inflammatory cytokine network (7). Persistent periodontal lesions may permit microbial components, modified autoantigens, and inflammatory mediators to enter the circulation, potentially contributing to chronic peripheral immune stimulation. These signals may influence the differentiation of Th17 and T follicular helper (Tfh) cells and modulate B-cell-supporting pathways involving B cell-activating factor (BAFF) and interleukin (IL)-21 (7, 8). Moreover, increased formation of neutrophil extracellular traps (NETs) has been observed in both periodontitis and SLE; the autoantigens and nucleic acid-protein complexes released by NETs can further activate plasmacytoid dendritic cells (DCs) and amplify the interferon (IFN)-α signaling pathway (9, 10). Nevertheless, whether these immune processes are simultaneously activated and functionally connected in patients with periodontitis–SLE comorbidity remains unclear.

Based on the evidence, this review summarizes the clinical associations and potential immunological crosstalk mechanisms between periodontitis and SLE, with particular emphasis on T–B cell crosstalk as a candidate convergent immune axis. We further examine whether periodontal inflammation may act as a peripheral amplifier of systemic autoimmunity, consider the potential reverse effects of SLE on periodontal homeostasis, and discuss the implications for therapeutic strategies and future research.

## 2 Clinical association between periodontitis and SLE

### 2.1 Epidemiological association between SLE and periodontitis

A growing body of studies, systematic reviews, and meta-analyses indicate that the prevalence of periodontitis in patients with SLE is significantly higher than that in the general population. A 2017 meta-analysis by Rutter-Locher et al. demonstrated that the risk ratio of periodontitis in patients with SLE was 1.76 (95% confidence interval (CI): 1.29–2.41), suggesting a significant epidemiological association between the two conditions (4). A 2024 systematic review by Tan et al. pointed out that the overall prevalence of periodontitis in patients with SLE was approximately 67% (95% CI: 57–77%), which was substantially higher than that in healthy controls (11). This high comorbidity was further confirmed by a nationwide cohort study by Bolstad et al. in 2022 that found that patients with SLE had approximately 1.8-fold higher odds of periodontitis than controls (5). However, some studies did not find any significant differences in specific periodontal clinical parameters (such as probing depth and clinical attachment loss). This inconsistency may be attributed to factors such as sample size, disease stage, use of immunosuppressive therapy, and differences in oral hygiene status (12). Moreover, the immune status and long-term medication use in patients with SLE may alter local inflammatory responses and modify periodontal manifestations. Therefore, these factors should be considered when interpreting epidemiological findings.

### 2.2 Associations between periodontitis severity and SLE disease activity

Some studies have reported a positive association between periodontitis severity and SLE disease activity. The systematic review by Hussain et al. summarized evidence suggesting that patients with SLE and periodontitis tend to have higher Systemic Lupus Erythematosus Disease Activity Index (SLEDAI) scores, more active inflammatory status, and elevated levels of serum inflammatory cytokines. However, these findings were predominantly derived from observational studies and cannot determine the direction of the association.

Evidence regarding the systemic effects of periodontal treatment in patients with SLE remains limited. Fabbri et al. reported that periodontal treatment was associated with improved periodontal parameters and a reduction in SLEDAI scores in a small group of patients receiving immunosuppressive therapy (6). Notably, patients with SLE generally receive immunosuppressants or glucocorticoids that not only reduce the inflammatory manifestations of periodontal tissues, but also alter the periodontal microecology and immune responses, thereby altering the association between disease activity and periodontal status (5, 6). Therefore, future studies should control for factors such as medication use and behavioral variables in their experimental designs to obtain more reliable conclusions.

Currently, whether periodontal therapy can ameliorate systemic inflammation or immune imbalance remains unclear. Evidence from clinical studies and systematic reviews suggests that nonsurgical periodontal treatment may reduce disease activity and selected inflammatory markers in patients with rheumatoid arthritis (RA) (13, 14). Although these discoveries are insightful, they are constrained by limitations, such as small sample sizes, short follow-up durations, and the absence of control groups, and cannot confirm causal inferences (13, 14). Moreover, findings obtained in RA cannot be directly extrapolated to SLE. From an immunological perspective, periodontal therapy may reduce the local inflammatory burden and decrease the systemic dissemination of microbial and inflammatory products, thereby attenuating persistent innate immune activation. Nevertheless, whether these effects produce clinically meaningful improvements in SLE disease activity remains uncertain. Large-scale, randomized studies with standardized periodontal and rheumatological outcomes and long-term follow-up are required.

### 2.3 Unresolved questions and evidence gaps

Epidemiological and clinical data support an association between periodontitis and SLE, but they do not establish its direction or causality (15) (**Figure 1**). Periodontitis may act as a peripheral amplifier of systemic immune activation, whereas SLE-associated immune dysregulation and its treatment may increase susceptibility to periodontal inflammation. Alternatively, the two conditions may coexist because of shared environmental, immunological, or genetic susceptibility factors.

**Figure 1:**
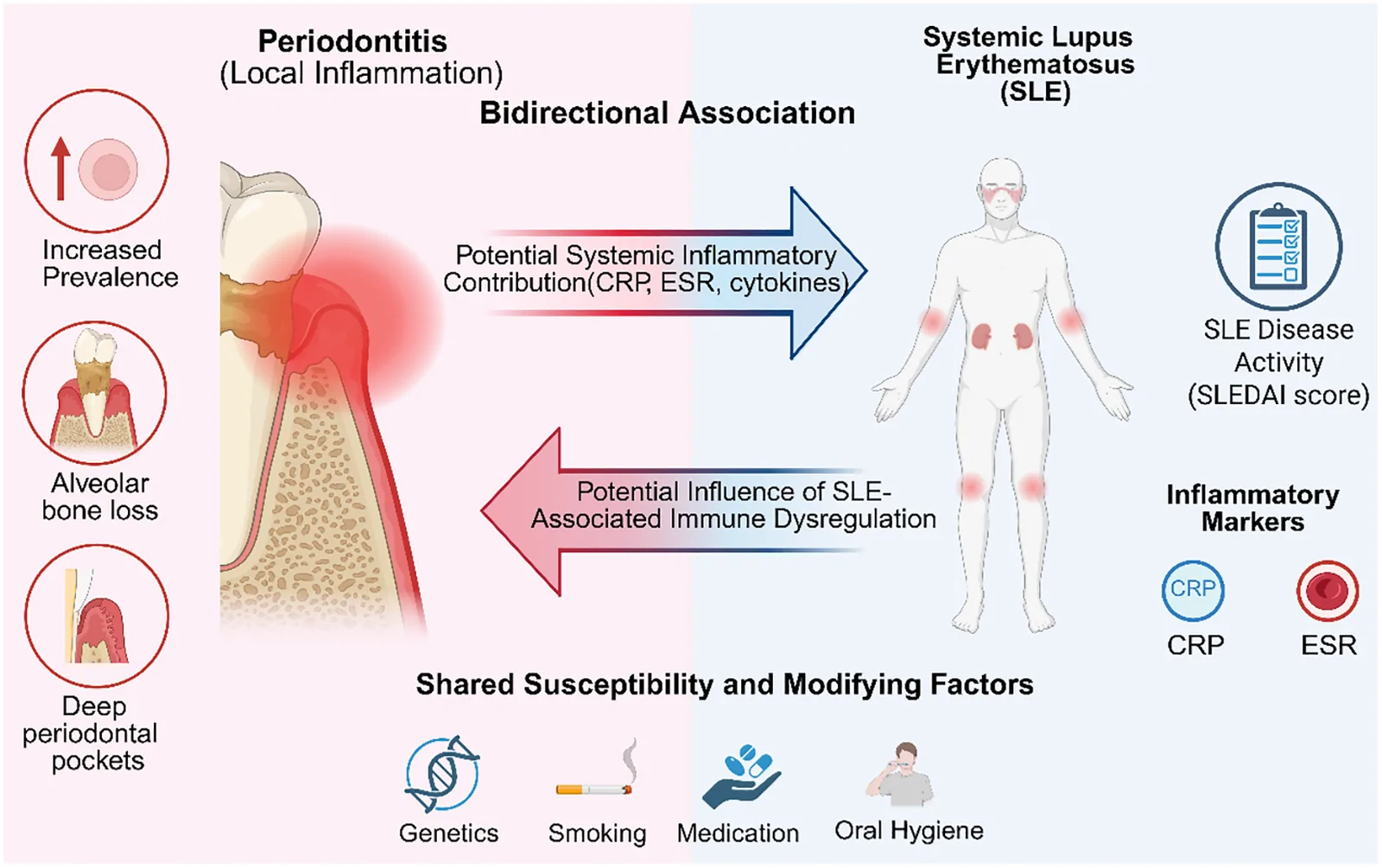
Bidirectional association between periodontitis and systemic lupus erythematosus (SLE). Epidemiological studies have reported a higher prevalence or odds of periodontitis in patients with SLE. Periodontal inflammation may be associated with increased systemic inflammatory burden, whereas SLE-associated immune dysregulation and treatment may modify periodontal susceptibility and severity. Potential shared susceptibility and modifying factors include genetics, smoking, oral hygiene, disease-related factors, and medication use. The figure illustrates a bidirectional association and does not establish causality.

Current evidence is insufficient to determine whether periodontitis independently contributes to the development of SLE, aggravates established disease activity, or represents a parallel manifestation of systemic immune dysregulation. Genetic analyses have suggested that genetically predicted circulating immune-cell traits, including neutrophil, natural killer T-cell, and plasmacytoid dendritic-cell traits, may be associated with periodontitis susceptibility (16). However, these findings do not demonstrate that periodontitis and SLE share specific causal genetic variants or a common genetic architecture. Furthermore, whether dendritic-cell activation, T–B cell dysregulation, cytokine networks, and NET-related pathways are simultaneously activated and functionally connected in patients with periodontitis–SLE comorbidity remains unknown. These questions require prospective cohorts, well-controlled periodontal intervention studies, and experimental comorbidity models.

## 3 Potential shared immunopathogenic mechanisms

### 3.1 Chronic inflammation induces the breakdown of immune tolerance

Periodontitis is a chronic inflammatory disease driven by complex microbial communities that can lead to sustained activation of the local immune system, systemic dissemination of inflammatory mediators, and induction of systemic low-grade inflammatory responses (3). Under prolonged inflammatory stress, antigen-presenting cell activation and maturation may be enhanced, as reflected by increased expression of co-stimulatory molecules and greater antigen-presenting capacity, potentially lowering the threshold for autoreactive lymphocyte activation (17). Persistent tissue destruction results in the continuous release of cellular components and modified proteins. These substances are more prone to undergo processing and structural alterations in the inflammatory microenvironment, thereby displaying novel antigenic characteristics and triggering the aberrant recognition and clonal expansion of T/B cells (18). Therefore, periodontitis represents a persistent peripheral source of microbial and inflammatory immune stimulation, which may amplify pre-existing systemic immune dysregulation in susceptible individuals. However, current evidence does not establish that periodontitis independently initiates systemic autoimmunity or SLE.

### 3.2 Microbial factors and induction of cross-reactivity and citrullinated antigens

Periodontitis is closely associated with a group of pathogenic bacteria known as the red complex, which includes *Porphyromonas gingivalis*, *Tannerella forsythia*, and *Treponema denticola*. These pathogens not only contribute to local tissue damage but may also influence systemic immune responses through several immunological mechanisms (19, 20). Among them, *Porphyromonas gingivalis* is a key bacterial species that has prominent systemic immune effects. It expresses P. gingivalis peptidylarginine deiminase, which promotes the citrullination of host and bacterial proteins (21). This process can alter protein structure and antigenicity, thereby generating citrullinated neoepitopes that can alter immune recognition. The contribution of citrullination to autoimmunity is best established in RA, where ACPA are characteristic. In SLE, direct evidence that periodontal inflammation-associated citrullination contributes to systemic autoimmunity is limited; therefore, any role in enhancing antigen immunogenicity or epitope spreading should currently be considered hypothetical (22). However, direct evidence supporting this mechanism in patients with periodontitis–SLE comorbidity remains limited. In addition, lipopolysaccharides, outer membrane vesicles, nucleic acid fragments, and metabolites derived from *P. gingivalis* may cross the inflamed gingival barrier or reach the systemic circulation, thereby triggering systemic activation of toll-like receptor (TLR) signaling (23). These molecular patterns can induce the maturation of DCs, upregulate the expression of co-stimulatory molecules, and enhance the capacity for self-antigen presentation, creating favorable conditions for T-cell autoreactivity (24).

Molecular mimicry has also been proposed as a potential cross-reactive mechanism. Sequence or structural similarities between microbial molecules and host proteins could permit cross-reactive recognition by antibodies or T-cell receptors. Although molecular mimicry has been implicated in several autoimmune diseases, direct evidence that periodontal pathogen-derived antigens initiate SLE-specific autoreactivity remains limited. Therefore, molecular mimicry should currently be regarded as a biologically plausible hypothesis rather than an established mechanism linking periodontitis to SLE (25).

Meanwhile, the subgingival microflora of patients with SLE exhibits significant dysbiosis, showing marked differences in community composition and decreased diversity compared to healthy controls. A study by Corrêa et al. found that the α-diversity of the subgingival microflora was reduced in patients with SLE, accompanied by elevated levels of potential proinflammatory cytokines, suggesting an association between local microbial disturbances and inflammatory status (26, 27). In addition, alterations in oral and intestinal microecology are associated with SLE disease activity in multiple observational studies, indicating that local microecological imbalances may be involved in systemic immune disorders through pathways such as immune stimulation and enhanced antigen presentation (28). In summary, periodontal microecological abnormalities provide a biologically plausible framework linking local inflammation with systemic immune dysregulation through citrullination, molecular mimicry, and microbial dysbiosis. However, their causal contribution to SLE remains to be established.

### 3.3 Cytokine networks and inflammatory amplification

A shared characteristic between periodontitis and SLE is the hyperactivation and imbalance of cytokine networks. The crosstalk between these immune signals primarily manifests through multiple key cytokine pathways.

#### 3.3.1 IL-6 signaling pathway

IL-6 is a candidate mediator linking periodontal inflammation with systemic immune activation. Stimulation by periodontal pathogens can induce IL-6 production by gingival fibroblasts, macrophages, and T cells (29). IL-6 is also known to promote B-cell differentiation and plasma-cell responses; however, whether periodontal IL-6 signaling directly contributes to systemic Tfh–B-cell dysregulation in SLE remains to be determined. In SLE, elevated IL-6 levels are closely correlated with anti-dsDNA antibody titers and disease activity (30). IL-6 can also facilitate the formation of Tfh cells and enhance their helper function toward B cells, thereby establishing a pathogenic Tfh-B cell axis (31). Nevertheless, direct evidence demonstrating that periodontal IL-6 signaling drives systemic Tfh–B-cell abnormalities in patients with SLE remains lacking.

#### 3.3.2 IL-17/Th17 signaling pathway

Th17 cells and their signature cytokine IL-17A are upregulated in both periodontitis and SLE (32). IL-17 promotes alveolar bone resorption by inducing the expression of receptor activator of nuclear factor-κB ligand (RANKL), stimulates neutrophil recruitment, and triggers the release of proteases, thereby exacerbating tissue destruction (33). In SLE, IL-17-producing double-negative T cells have been identified in renal infiltrates from patients with lupus nephritis (34). In addition, IL-23/IL-17 axis is enhanced in chronic periodontitis, and related inflammatory pathways are closely associated with disease progression; clinically, significantly elevated levels of IL-17A and its receptor have been detected in the periodontal tissues of patient samples (35). These parallel abnormalities identify the IL-17/Th17 axis as a convergent inflammatory feature of periodontitis and SLE.

#### 3.3.3 BAFF/a proliferation-inducing ligand family signaling

BAFF and APRIL are key cytokines that maintain B cell survival and plasma cell generation. Serum BAFF levels are generally elevated in patients with SLE and are associated with autoantibody titers and disease activity (36). Increased BAFF and APRIL levels have also been detected in the gingival crevicular fluid or serum of patients with periodontal disease (37), supporting BAFF/APRIL signaling as a convergent feature of B-cell regulation in periodontitis and SLE. Whether periodontal BAFF/APRIL production contributes to systemic B-cell hyperactivation or SLE activity remains to be determined. Therefore, BAFF/APRIL signaling should be considered a candidate shared pathway rather than an established causal link between the two diseases.

#### 3.3.4 Other inflammatory cytokines

Tumor necrosis factor-α (TNF-α), IL-1β, IL-23, and type I interferons (e.g., IFN-α/β) are highly active in both diseases. In particular, IFN-I signaling plays a central role in SLE pathogenesis; it induces the expression of genes involved in antigen presentation and autoantibody production (38). Altered type I interferon signaling has also been demonstrated in experimental periodontal disease. In a murine model induced by P. gingivalis, elevated type I interferon signaling was associated with local immune dysfunction and increased bone resorption, whereas blockade of this pathway improved local immune homeostasis and reduced bone loss (39). These findings support a role for type I interferons in periodontal inflammation. Overall, cytokine abnormalities in periodontitis and SLE may contribute to overlapping inflammatory environments. However, the available findings are primarily derived from separate studies of the two diseases, and whether these pathways are simultaneously activated and functionally connected in patients with periodontitis–SLE comorbidity remains to be determined.

### 3.4 Dysregulated immune regulation and positive feedback amplification

Persistent inflammation in periodontal disease may contribute to a hypothesized local–systemic immune feedback loop. Chronic periodontal lesions can activate DCs, T cells, and B cells, promoting the production of inflammatory cytokines and antibodies. Subsequent tissue injury may release additional antigens and inflammatory mediators, thereby sustaining local immune activation and potentially increasing the systemic inflammatory burden. In genetically or immunologically susceptible individuals, this process may amplify pre-existing systemic immune dysregulation (40). Conversely, SLE-associated immune abnormalities and immunosuppressive treatment may alter periodontal immunity and microbial homeostasis, thereby increasing susceptibility to persistent periodontal inflammation. Thus, the proposed feedback relationship may be bidirectional rather than a unidirectional effect of periodontitis on SLE (**Figure 2**). In addition, multiple non-classical pathological pathways, such as the crosstalk between inflammation and metabolic processes, oxidative stress, mitochondrial dysfunction, and endothelial cell dysregulation, have also been implicated in both periodontitis and SLE (41). Through their effects on cellular metabolism, vascular function, and inflammatory signaling, these processes may contribute to the maintenance of chronic inflammatory environments. However, their proposed bridging roles remain incompletely defined and have not been directly demonstrated in periodontitis–SLE comorbidity models.

**Figure 2:**
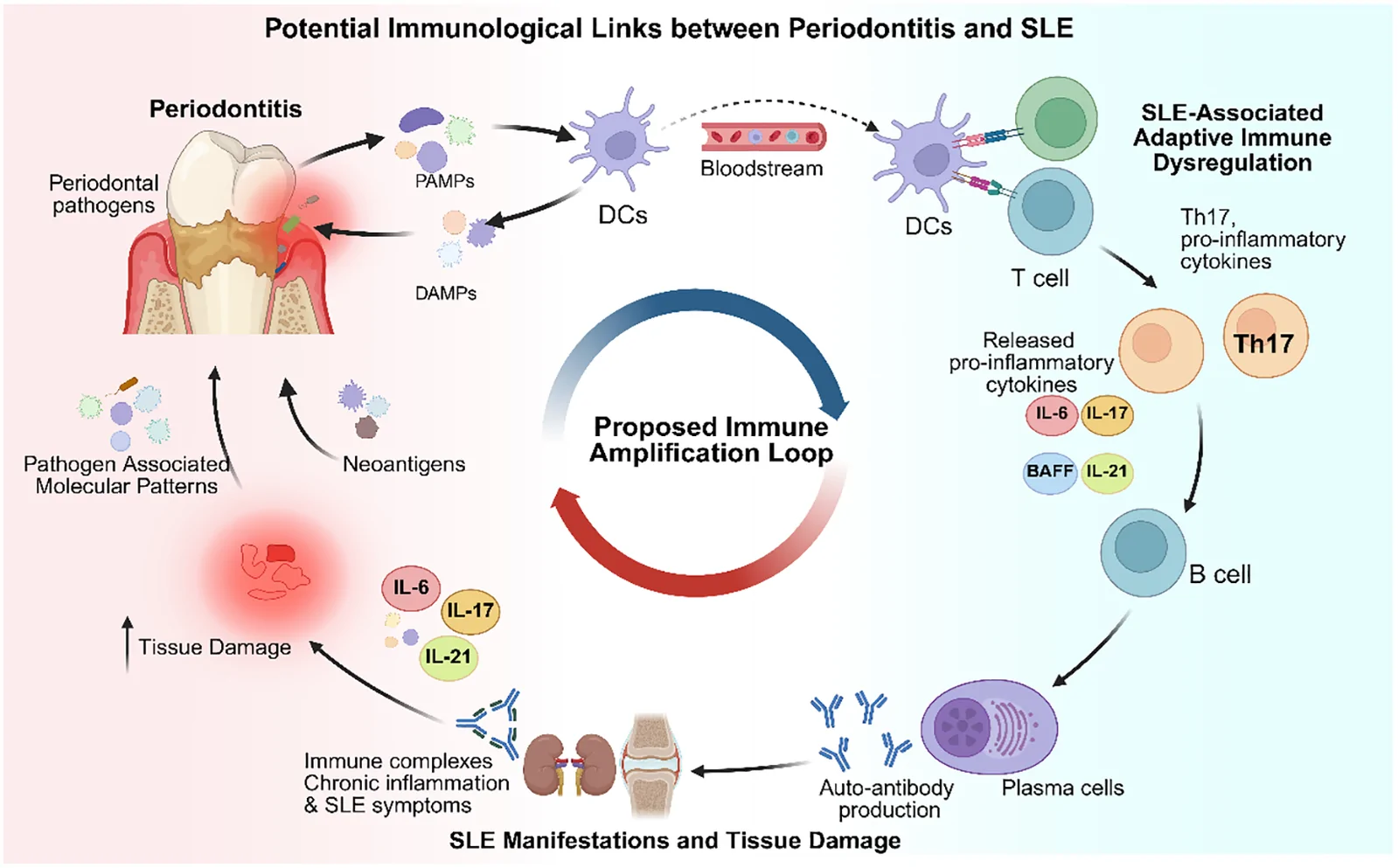
Potential shared immunopathogenic features of periodontitis and SLE. Periodontitis is characterized by PAMP/DAMP-associated dendritic-cell activation, inflammatory cytokine production, and tissue injury, whereas SLE involves dysregulated adaptive immune responses, B-cell and plasma-cell activation, autoantibody production, and immune-complex-mediated tissue damage. These parallel immunological abnormalities may provide a mechanistic framework for oral–systemic immune crosstalk; however, their coordinated operation in periodontitis–SLE comorbidity and any causal contribution of periodontal inflammation to SLE progression remain to be established. Dashed arrows indicate proposed cross-disease links rather than established causal relationships.

## 4 T and B cell crosstalk as a potential convergent immunological axis

Crosstalk between T and B cells is a key immunoregulatory mechanism in periodontitis and SLE. The two diseases exhibit overlapping, although not identical, immunopathological features involving T-cell subset imbalance, B-cell activation, and altered T–B cell interactions (12, 26).

### 4.1 T cell abnormalities: Th17/Treg imbalance and enhanced Tfh cell activity

An increased Th17/Treg ratio has been reported in both periodontitis and SLE, although the magnitude and consistency of this alteration may vary according to disease activity, sample type, and treatment status (42, 43). Increased Th17 responses can amplify local inflammation, promote tissue damage, and influence B-cell activation through mediators such as IL-17 (44). Increased frequencies or enhanced activity of Tfh cells have also been reported in both diseases. Through IL-21 and CD40L signaling, Tfh cells support germinal center reactions, B-cell differentiation, and antibody production (46, 47). In SLE, excessive Tfh-cell activity contributes to autoreactive B-cell activation and pathogenic autoantibody production. In periodontitis, Tfh-associated responses may enhance local B-cell and plasma-cell activity; however, their contribution to systemic autoreactive humoral responses remains unclear (45, 46).

### 4.2 B cell abnormalities: expansion of memory B cells and plasma cells

Dysregulation of B-cell lineage differentiation is an important feature of both diseases. Increased numbers of activated B cells, memory B cells, and plasma cells have been reported in periodontal lesions (47). In SLE, autoreactive B-cell populations, altered memory B-cell compartments, and expanded antibody-secreting cell populations have been observed (48, 49). In periodontitis, these alterations contribute primarily to local antibody responses and inflammatory tissue injury, whereas in SLE they promote pathogenic autoantibody production and immune-complex formation. Thus, B-cell activation represents an overlapping feature of the two diseases, although the antigen specificity and pathological consequences of the antibody responses differ (49).

### 4.3 T-B cell crosstalk: commonality of the Tfh-IL-21 axis and BAFF pathway

Dysregulated T–B cell crosstalk represents a convergent immunological network in periodontitis and SLE. In SLE, the Tfh-IL-21 axis is abnormally activated. IL-21, a major effector cytokine produced by Tfh cells, can promote B-cell proliferation, germinal center responses, somatic hypermutation, plasma-cell differentiation, and antibody secretion (50, 51).

Multiple clinical studies have shown that IL-21 levels are elevated in patients with chronic periodontitis, which is observed in the serum, saliva, periodontal tissues, and gingival crevicular fluid, and is positively correlated with inflammatory severity and clinical parameters (e.g., probing depth and attachment loss) (52–55). Furthermore, IL-21 is secreted by inflammatory T cells (e.g., Th17 cells) and participates in the proinflammatory network. Collectively, available evidence indicates increased IL-21 signaling within the periodontal inflammatory environment. However, whether IL-21 elevation in periodontitis reflects a Tfh-driven mechanism of B-cell dysregulation analogous to that observed in SLE requires direct experimental validation (53).

The BAFF pathway represents a candidate convergent pathway regulating B-cell survival in periodontitis and SLE. As a member of the TNF superfamily, BAFF is a critical regulatory factor for B cell survival and maturation. Its overexpression can promote the survival of autoreactive B cells, plasma cell maintenance, and autoantibody production and is closely associated with SLE disease activity (56). Clinical observations have shown elevated soluble BAFF levels in the serum of patients with SLE that are positively correlated with autoantibody levels and disease activity indices (57). BAFF-targeted therapy, particularly the anti-BAFF monoclonal antibody belimumab, has demonstrated clinical efficacy in reducing disease activity and the risk of severe flares in selected patients with SLE (58, 59). This clinical efficacy supports the pathogenic importance of BAFF-dependent B-cell survival in SLE, although it does not establish that periodontal BAFF signaling contributes to systemic disease activity.

Similarly, increased BAFF expression or concentrations have been reported in gingival tissues, gingival crevicular fluid, or serum from patients with periodontitis, with some studies reporting associations with periodontal clinical parameters (60, 61). These findings suggest that BAFF may contribute to local B-cell survival and inflammatory responses in periodontal tissues. However, it remains unclear whether periodontal BAFF production has sufficient systemic effects to alter autoreactive B-cell responses or SLE disease activity. Thus, BAFF signaling represents an important point of convergence in B-cell regulation between the two diseases, although its functional contribution to periodontitis–SLE comorbidity remains to be established.

Recent cross-disease bioinformatics studies provide additional support for positioning T–B-cell dysregulation as a candidate convergent axis between periodontitis and SLE. Zhao et al. identified 50 shared candidate genes enriched in the PI3K–Akt and B-cell receptor signaling pathways, with the B-cell-associated molecules CD19 and CD79A occupying central positions in the interaction network. Immune-deconvolution analysis further identified disease-specific immune-cell alterations, including decreased Tfh-cell and increased plasma-cell infiltration in the periodontitis dataset (62). Wang et al. identified 32 crosstalk genes enriched in NF-κB, IL-17, and cytokine-related pathways and linked these genes to altered T- and B-cell infiltration (63). Cheng et al. identified 21 overlapping co-expression genes, while single-cell analysis localized key molecular signals to T-cell and antigen-presenting-cell populations and associated them with plasma-cell-related immune patterns (64). Although these studies were based primarily on independent periodontitis and SLE datasets and did not directly demonstrate Tfh–B-cell contact or BAFF/IL-21-mediated intercellular signaling, their convergence on antigen presentation, T-cell activation, B-cell receptor signaling, and plasma-cell responses provides hypothesis-generating molecular support for the T–B-cell-centered framework proposed here. These pioneering analyses therefore identify specific cellular and signaling nodes for future validation in comorbid patient samples, T–B-cell co-culture systems, and experimental animal models.

### 4.4 Crosstalk among other immune cells

Beyond T–B cell crosstalk, both SLE and periodontitis exhibit overlapping abnormalities involving other immune-cell populations. These include altered DC activation and antigen-presenting functions (24, 65), altered macrophage polarization (66, 67), and dysregulated NET formation (68). NET-associated DAMPs, including HMGB1, may further stimulate innate immune signaling and influence adaptive immune responses (69). These processes may interact with T–B-cell dysregulation and sustain chronic inflammation; however, their synergistic operation in periodontitis–SLE comorbidity has not been directly demonstrated.

### 4.5 Periodontitis as a potential peripheral amplifier of systemic autoimmunity

Chronic periodontitis has been proposed to act as a peripheral amplifier of systemic immune activation through several potentially interacting pathways (70). Epidemiological studies have consistently reported a higher prevalence or risk of periodontitis among patients with SLE (4, 20, 70). This finding supports an association between the two diseases but does not establish that periodontitis precedes or promotes SLE. Some observational studies have also examined associations between a history of periodontitis and subsequent SLE diagnosis. However, these findings remain susceptible to residual confounding, reverse causation, surveillance bias, and differences in medication use and healthcare-seeking behavior. At the mechanistic level, persistent release of PAMPs and DAMPs from periodontal lesions can activate pattern-recognition receptors and antigen-presenting cells, including DCs. This inflammatory environment can favor the differentiation and activation of proinflammatory CD4^+^ T-cell populations, including Th17 and Tfh cells, and may thereby increase the systemic inflammatory burden (71). The periodontal inflammatory environment is also associated with increased expression of B-cell survival and differentiation factors, including BAFF, APRIL, and IL-21, which support local B-cell and plasma-cell responses (72, 73). Increased NET formation has been associated with periodontal inflammation and tissue damage. In SLE, impaired NET clearance and NET-derived nucleic acid–protein complexes can activate pDCs and type I interferon signaling. Together, these parallel findings identify the NET–pDC–IFN-I axis as another candidate pathway of oral–systemic immune crosstalk (74, 75) (**Figure 3**).

**Figure 3:**
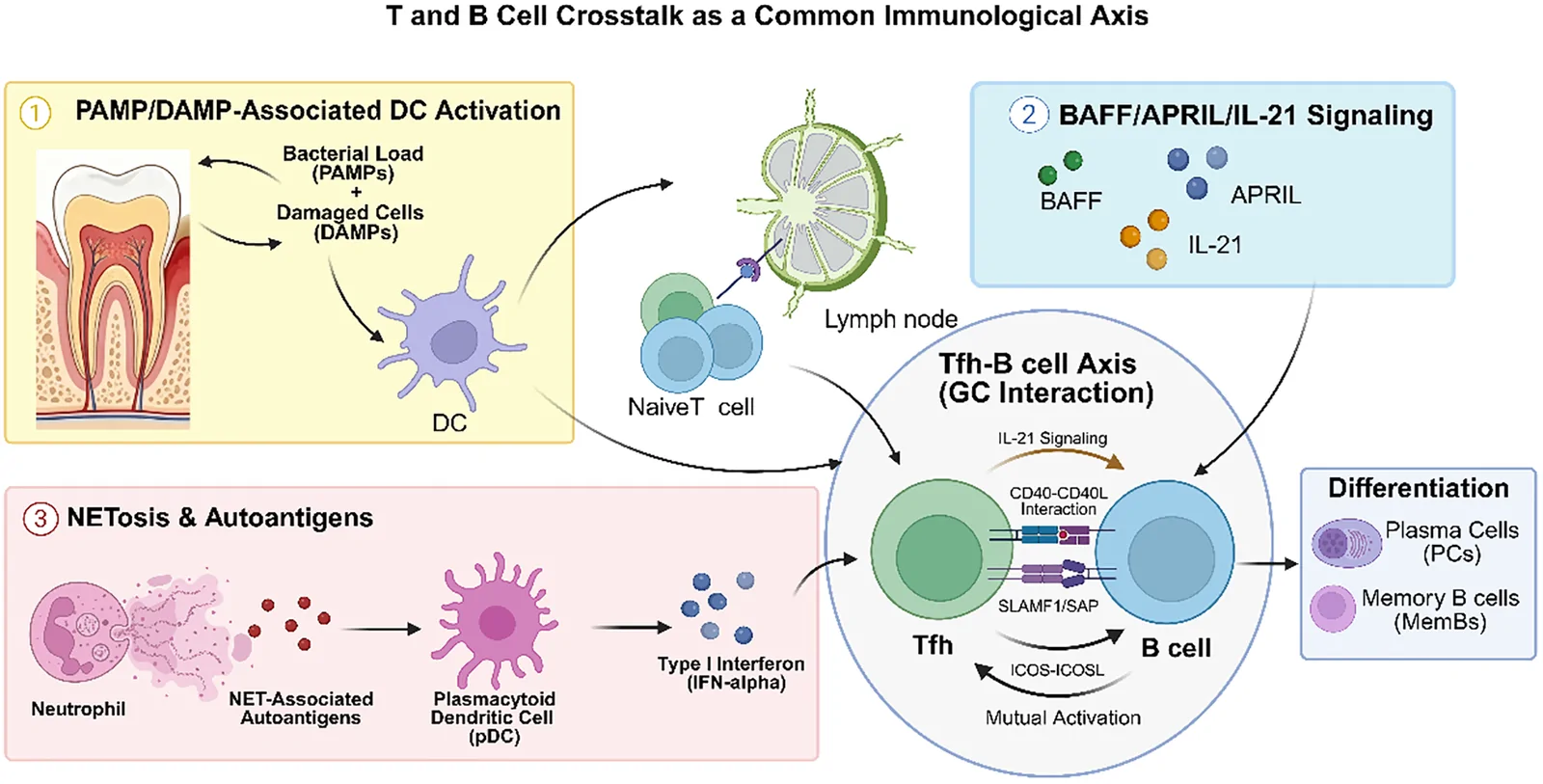
T–B-cell crosstalk as a potential convergent immune axis in periodontitis and SLE. PAMP/DAMP-associated dendritic-cell activation, BAFF/APRIL/IL-21 signaling, and NET–pDC–type I interferon responses may converge on Tfh–B-cell interactions and B-cell differentiation. These pathways are supported largely by parallel evidence from periodontitis and SLE, whereas their direct functional coupling in periodontitis–SLE comorbidity remains to be established.

Collectively, three interrelated processes may contribute to peripheral immune stimulation associated with periodontitis: (1) sustained exposure to microbial antigens and PAMPs/DAMPs; (2) increased expression of B-cell-supporting cytokines, including BAFF, APRIL, and IL-21; and (3) enhanced NET formation and autoantigen exposure. Together, these processes provide a biologically plausible framework for the amplification of pre-existing systemic immune dysregulation by chronic periodontal inflammation. Nevertheless, direct evidence that periodontitis initiates SLE or independently increases SLE disease activity remains insufficient. Conversely, SLE-associated immune dysregulation may alter periodontal immune homeostasis and the host response to subgingival microbiota. Importantly, recent experimental evidence from an MRL-lpr ligature-induced SLE–periodontitis model showed increased periodontal inflammation and bone resorption in the SLE background (76). These findings provide direct support for the reverse SLE-to-periodontitis direction *in vivo*, although validation in independent models and longitudinal human cohorts is still required.

## 5 Therapeutic and research implications

### 5.1 Potential benefits of periodontal therapy for systemic inflammation

Periodontal therapy effectively improves local periodontal inflammation and may reduce selected systemic inflammatory markers; however, evidence that periodontal treatment improves SLE disease activity remains preliminary. Clinical studies suggest that non-surgical periodontal therapy may reduce selected circulating inflammatory markers, particularly CRP, although the magnitude and consistency of its effects on IL-6, TNF-α, metabolic parameters, and vascular outcomes vary across populations and study designs (77, 78). In a small controlled study, Fabbri et al. reported improvements in periodontal parameters and a reduction in SLEDAI scores after periodontal treatment in patients receiving immunosuppressive therapy (6). More recently, Pessoa et al. reported a significant reduction in plasma high-sensitivity C-reactive protein 30 days after nonsurgical periodontal treatment in 38 patients with SLE and periodontitis (79). However, the small sample size, short follow-up period, and concomitant immunosuppressive treatment limit causal interpretation. These preliminary findings suggest that periodontal treatment may have value as an adjunctive component of oral healthcare in patients with SLE, but they do not establish periodontal therapy as a disease-modifying treatment for SLE (**Figure 4**). From a clinical perspective, periodontal screening and timely periodontal care can be incorporated into the multidisciplinary management of patients with SLE because they improve oral health and reduce local inflammatory burden. However, current evidence is insufficient to recommend periodontal treatment specifically for controlling SLE activity or preventing SLE flares.

**Figure 4:**
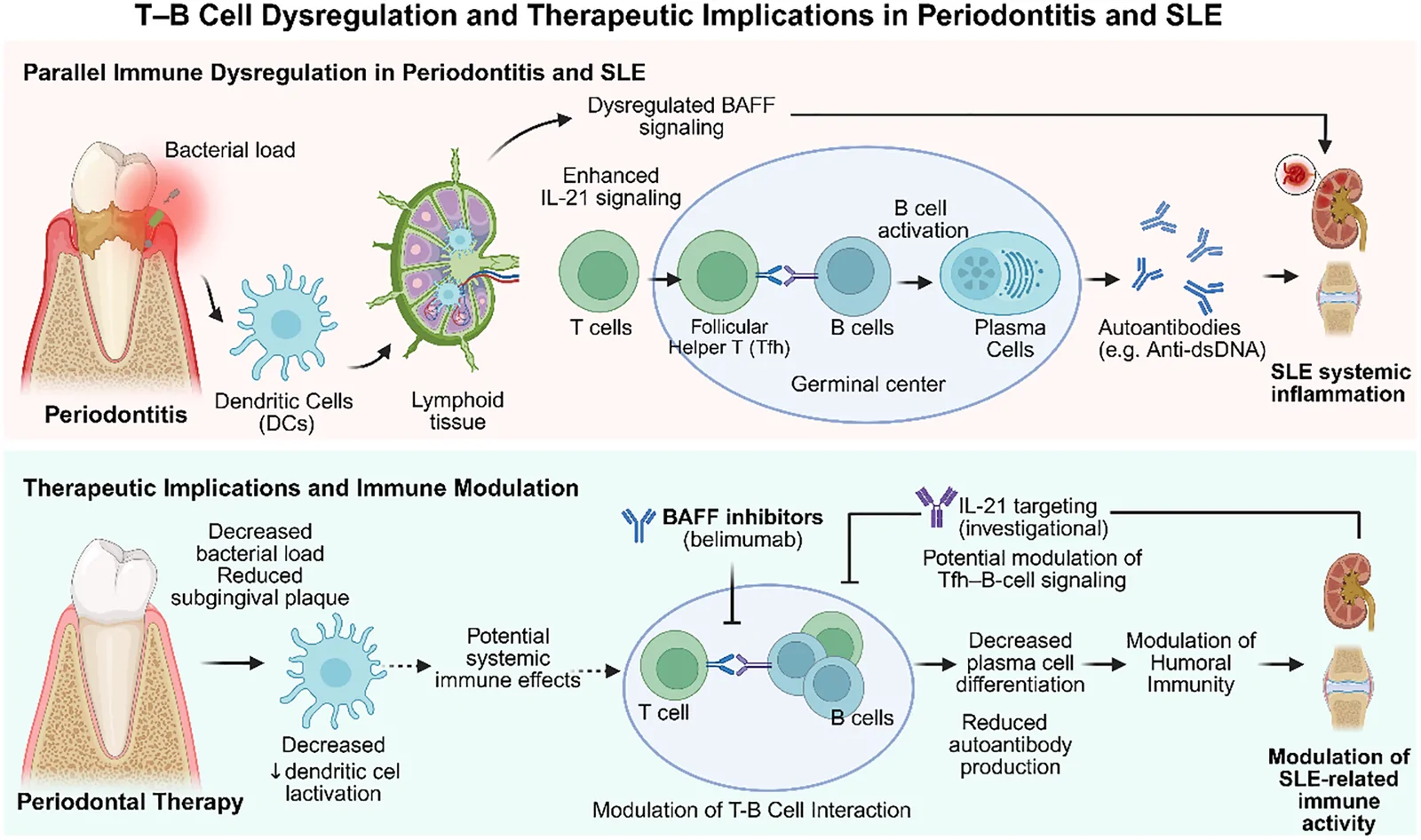
Therapeutic implications of T–B-cell dysregulation in periodontitis and SLE. Upper panel: Parallel immune abnormalities reported in periodontitis and SLE include dendritic-cell activation, altered Tfh responses, and dysregulated BAFF/IL-21 signaling, which may support B-cell activation in their respective disease contexts. Lower panel: Periodontal therapy reduces local periodontal inflammatory burden, whereas BAFF-targeted therapy has an established role in SLE and IL-21-directed approaches remain investigational. Whether periodontal treatment modifies systemic Tfh–B-cell responses, BAFF/IL-21 signaling, autoantibody production, or SLE-related immune activity remains to be established.

Reducing periodontal microbial and inflammatory burden may decrease local innate immune activation. *Porphyromonas gingivalis* expresses *P. gingivalis* peptidylarginine deiminase, which can promote protein citrullination, and periodontal inflammation may enhance neutrophil activation and NET formation (21, 80). In SLE, NET-derived nucleic acid–protein complexes can activate plasmacytoid DCs and type I interferon signaling (75, 81). These parallel observations provide a mechanistic rationale for investigating whether periodontal treatment influences the systemic NET–pDC–IFN-I axis. However, such an effect has not yet been directly demonstrated.

### 5.2 Therapeutic implications of targeting T–B cell dysregulation

The Th17/IL-17, Tfh–IL-21, BAFF, and NET-related pathways offer plausible therapeutic entry points because they are altered in both periodontitis and SLE. Whether intervention at any of these nodes can improve periodontal and systemic outcomes simultaneously remains unknown.

#### 5.2.1 IL-17 pathway

IL-17 contributes to osteoclastogenesis and inflammatory tissue destruction in periodontitis and has also been implicated in renal and systemic inflammation in SLE. Nevertheless, biological plausibility has not translated into established clinical efficacy in SLE. In the phase III SELUNE trial, secukinumab did not demonstrate superior efficacy over placebo in patients with active lupus nephritis, and the study was terminated early following a futility analysis (82). Therefore, IL-17 inhibition should currently be regarded as an exploratory rather than a validated therapeutic strategy for periodontitis–SLE comorbidity.

#### 5.2.2 BAFF-targeted therapy

BAFF is a central regulator of B-cell survival and autoreactive humoral responses in SLE. Belimumab, a monoclonal antibody targeting soluble BAFF, has demonstrated clinical efficacy in reducing SLE disease activity and severe flares in randomized controlled trials (83). These findings validate BAFF-dependent B-cell survival as a therapeutic target in SLE. Increased BAFF expression has also been reported in periodontal tissues; however, there is currently no evidence that belimumab or other BAFF-targeted agents improve periodontal outcomes. Future clinical studies of BAFF-targeted therapy in SLE could incorporate standardized periodontal assessments and measurements of local and circulating BAFF to determine whether treatment responses extend to the periodontal compartment.

#### 5.2.3 Tfh–IL-21 axis

The Tfh–IL-21 axis represents another potential therapeutic node directly related to T–B cell dysregulation. In SLE, excessive IL-21 signaling can promote germinal center responses, autoreactive B-cell differentiation, and autoantibody production. Elevated IL-21 levels have also been reported in patients with periodontitis, although their cellular sources and systemic consequences remain incompletely defined (50–54). Therapeutic inhibition of IL-21 or IL-21R signaling remains investigational, and there is currently no evidence that it simultaneously improves periodontal inflammation and SLE activity. Future studies should determine whether changes in circulating and periodontal Tfh cells, IL-21, B-cell subsets, and autoantibody profiles occur in parallel and whether this axis is modified by periodontal treatment or pathway-specific intervention.

### 5.3 Future research priorities: mechanistic, clinical, and translational directions

Conventional histology provides spatial context but has limited multiplexing capacity, whereas flow cytometry enables detailed cellular phenotyping but largely loses spatial information. Integrating these approaches with longitudinal clinical sampling, experimental models, and multi-omics technologies may provide a more comprehensive understanding of oral–systemic immune crosstalk.

#### 5.3.1 Prospective clinical cohorts and periodontal intervention studies

Large, prospective cohorts are needed to determine the temporal relationship between periodontitis and SLE onset, activity, and flares (26). Repeated assessments of periodontal status, SLE disease activity, medication exposure, smoking, oral hygiene, metabolic factors, and systemic inflammatory markers should be incorporated to reduce confounding and clarify directionality. Building on preliminary evidence that periodontal treatment may improve SLE disease activity (6), well-powered randomized periodontal intervention studies should evaluate standardized periodontal outcomes together with SLEDAI scores, flare rates, autoantibody profiles, complement levels, and circulating Tfh–B-cell, BAFF, and IL-21 measurements. Stable background immunosuppressive treatment and sufficiently long follow-up will be essential for interpreting systemic effects.

#### 5.3.2 Experimental comorbidity models and causal perturbation

Mechanistic studies should establish experimental models in which periodontitis is induced in lupus-prone animals and, conversely, periodontal responses are examined during the development of systemic autoimmunity (76). Such models would allow temporal assessment of whether periodontal inflammation precedes or amplifies systemic immune abnormalities. Pathway-specific interventions targeting Tfh cells, IL-21, BAFF, NET formation, or type I interferon signaling could then be used to determine whether these mechanisms are causally involved in both periodontal and systemic outcomes (84–86).

#### 5.3.3 Single-cell and immune-repertoire analyses

Single-cell sequencing has identified key pathological cell populations in SLE, including Tfh cells, age-associated B cells, and IFN-highly responsive cell populations (87–90). Applying this technology to periodontal tissues and peripheral blood may help define the states and interaction networks of Th17 cells, Tfh cells, B cells, plasma cells, DCs, and NET-associated neutrophil populations. Integration with T-cell receptor and B-cell receptor repertoire sequencing may further determine whether clonally related lymphocyte populations are present in oral and systemic compartments. Because NET formation cannot be fully characterized by transcriptomics alone, these analyses should be combined with imaging and protein-based measurements of NET-associated components.

#### 5.3.4 Spatial multi-omics and tissue organization

Spatial transcriptomics can reveal the spatial tissue architecture of immune cells at the *in-situ* tissue level, providing a pivotal approach for dissecting intercellular crosstalk within the inflammatory microenvironment. In various chronic inflammatory, autoimmune, and tumor tissues, ectopic lymphoid structures (ELSs) are spatially organized immune cell aggregates analogous to germinal centers that play critical roles in antigen presentation and immune amplification (91).

Spatial omics has been employed to identify and characterize the distribution and cellular composition of such structures under distinct pathological conditions (92). Meanwhile, the formation of tertiary lymphoid structure (TLS)-like structures has been observed in oral-related chronic inflammatory conditions such as pulpitis, suggesting that the potential existence of similar ectopic lymphoid tissues in periodontitis warrants further investigation using spatial functional omics approaches (93).

#### 5.3.5 Translational and clinical integration

Future translational studies should evaluate whether periodontal status modifies responses to SLE-directed therapies and whether SLE treatments alter periodontal inflammation, microbial ecology, or tissue repair. Standardized periodontal screening in SLE cohorts and the inclusion of oral outcomes in trials of established BAFF- and type I interferon-targeted SLE therapies, as well as future IL-21-targeted strategies (85, 86, 94), would help determine the clinical relevance of the oral–systemic immune axis. Until such evidence becomes available, periodontal therapy should be recommended for improving oral health rather than as a substitute for established SLE treatment.

Candidate biomarker panels integrating circulating Tfh and B-cell subsets, BAFF, IL-21, NET-related markers, and standardized periodontal parameters should be evaluated for patient stratification and treatment-response monitoring in patients with periodontitis–SLE comorbidity (71, 72, 75). Because many of these immune markers are not disease-specific, their predictive and discriminative value will require validation in well-characterized prospective cohorts.

## 6 Conclusion

Current evidence supports an association between periodontitis and SLE, but does not establish a shared causal pathway (4, 20). Their immunological overlap includes dysregulated T–B-cell responses, BAFF/IL-21 signaling, and NET-related inflammation. These pathways offer plausible routes by which periodontal inflammation could add to systemic immune activation, while SLE-associated immune dysfunction and treatment may influence periodontal homeostasis (95, 96). Cross-disease bioinformatics analyses lend hypothesis-generating support to this framework, but functional evidence in comorbid settings is still needed (62–64). Small interventional studies show improvements in oral health and suggest possible reductions in selected systemic inflammatory measures; a disease-modifying effect on SLE has not been established (6, 79). Periodontal care should therefore complement standard SLE management rather than replace it. Progress now depends on prospective cohorts, adequately powered intervention trials, comorbidity models, and pathway-specific experiments integrated with single-cell and spatial analyses. Together, these approaches can clarify directionality and determine whether the oral–systemic immune axis has value for risk stratification or coordinated care.
